# Towards the development of a wearable feedback system for monitoring the activities of the upper-extremities

**DOI:** 10.1186/1743-0003-11-2

**Published:** 2014-01-08

**Authors:** Zhen G Xiao, Carlo Menon

**Affiliations:** 1 MENRVA Group, School of Engineering Science, Faculty of Applied Science, Simon Fraser University, 8888 University Drive, Burnaby, BC V5A 1S6, Canada

**Keywords:** Force myography, Classification, Extreme learning machine, Constraint-induced movement therapy, Functional exercise

## Abstract

**Background:**

Body motion data registered by wearable sensors can provide objective feedback to patients on the effectiveness of the rehabilitation interventions they undergo. Such a feedback may motivate patients to keep increasing the amount of exercise they perform, thus facilitating their recovery during physical rehabilitation therapy. In this work, we propose a novel wearable and affordable system which can predict different postures of the upper-extremities by classifying force myographic (FMG) signals of the forearm in real-time.

**Methods:**

An easy to use force sensor resistor (FSR) strap to extract the upper-extremities FMG signals was prototyped. The FSR strap was designed to be placed on the proximal portion of the forearm and capture the activities of the main muscle groups with eight force input channels. The non-kernel based extreme learning machine (ELM) classifier with sigmoid based function was implemented for real-time classification due to its fast learning characteristics. A test protocol was designed to classify in real-time six upper-extremities postures that are needed to successfully complete a drinking task, which is a functional exercise often used in constraint-induced movement therapy. Six healthy volunteers participated in the test. Each participant repeated the drinking task three times. FMG data and classification results were recorded for analysis.

**Results:**

The obtained results confirmed that the FMG data captured from the FSR strap produced distinct patterns for the selected upper-extremities postures of the drinking task. With the use of the non-kernel based ELM, the postures associated to the drinking task were predicted in real-time with an average overall accuracy of 92.33% and standard deviation of 3.19%.

**Conclusions:**

This study showed that the proposed wearable FSR strap was able to detect eight FMG signals from the forearm. In addition, the implemented ELM algorithm was able to correctly classify in real-time six postures associated to the drinking task. The obtained results therefore point out that the proposed system has potential for providing instant feedback during functional rehabilitation exercises.

## Background

Body motion and physiological data registered by wearable sensors have been used for diagnostics, as well as monitoring the rehabilitation progress of people recovering from an injury or living with a chronic disease such as stroke [1]. These data can provide objective feedback on patient’s health status and the progress of rehabilitation, which allow therapists to optimize the rehab routine [2]. Feedback to the patient should be provided in real-time, as there is evidence that instant feedback can further motivate the user to reach the targeted goal or even to keep increasing the amount of exercise [3]. An example of monitoring device that provides instant feedback to the user is the pedometer, which counts the number of steps and effectively motivates people to increase their walking activities towards better health [4]. However, compared to the lower-extremities activity monitoring, there are few affordable and easy to use devices that can provide instant feedback of the activities of the upper-extremities to motivate the patients. Many studies have shown the increase of upper-extremities activity can lead to better outcomes after neurological conditions including stroke [5], head injury [6], incomplete spinal cord injury [7] and cerebral palsy [8]. Thus, there are great needs for having such a device for providing instant feedback of targeted rehab exercise that involves upper-extremities movement.

Current commercial wrist accelerometers, such as the Actical [9] and the Actigraph [10], can provide objective measures of arm use based on multidirectional acceleration data of the upper-extremities. However, these systems provide no real-time feedback to the user, nor are able to capture any information about hand use, which is one of the most important upper-extremities functions in our daily life. Besides the use of accelerometers, there are other methods available for capturing both the hand and arm movement. One example is the use of a data-glove, such as the Cyberglove [11], which incorporates both inertial measurement unit (IMU) sensors and flexible bend sensors for motion capturing. However, data-gloves are generally designed for virtual reality applications that require the use of a host CPU. Moreover, data-gloves limit the tactile sensation of the user’s fingers, thus limiting the effectiveness of rehabilitation protocols involving the somatosensory system.

In addition to commercially available devices, current active research focuses on processing bio-signals through the use of surface electromyography (sEMG) to predict the upper-extremities movements that involve elbow, wrist or/and hand [12-14]. Even though this method frees the hand and allows full tactile sensation, it requires expensive and sizable equipment, as well as high-level signal processing for feature extraction. This approach is therefore not very suitable for inexpensively detecting movements in outdoor activities or in the home environment.

Other than using accelerometer, data acquisition glove or sEMG for monitoring the upper extremity movement, there is a relatively unexplored method named force myography (FMG). FMG is referred to a technique which use force sensor to capture the expansion/contraction of the large surface muscle [15]. The use of FMG to distinguish limb movements was preliminarily explored by O.Amft et al. [16] who used two force resistive sensors (FRS) on the forearm, and were able to visually distinguish four types of arm gestures on a data plot. The use of FMG was also investigated for monitoring cycling activity by placing FSRs on the upper leg [17]. The research performed by G. Ogris [18], X. Wang [19] and Li et al. [20] showed the possibility to predict different arm and finger gestures by using multiple FSRs pressed against the arm. While their methodologies did not allow having a wearable system for real-time feedback, these works proved the feasibility of using FMG for monitoring upper-extremities gestures.

In this paper, we propose a novel system to detect different upper-extremities postures in real-time through the use of a lightweight and wearable forearm FSR strap. The strap has multiple FSR sensors, whose signals are classified in real-time to distinguish different upper-extremities postures. The FRS strap was conceived to be easy to use by a layperson. Location of the single forearm muscle groups is therefore not required every time the sensor strap is worn. The FRS strap was also designed to be a standalone device, which does not require any external equipment, such as a powerful computer or auxiliary sensors, for its calibration. The strap can therefore be used in unstructured environments, such as the patient’s home.

Among the different existing classifiers, we utilized the Extreme Learning Machine (ELM) for processing signals of our FSR strap system. The ELM was first proposed by G.Huang et.al [21] in 2004, and has been refined since. In recent publications, ELM has been shown to have equal or superior performance compared to the popular Support Vector Machine (SVM) [22] and Artificial Neural Network (ANN) [23] for supervised multiclass classification, but with simpler architecture and faster learning speed [24,25]. Simple architecture and fast learning speed are crucial for our system. It should in fact be noted that in order to have an affordable and lightweight device to monitor the upper-extremities activity, a low power and low profile microcontroller would to be used. Due to the potentially low computational power available, the simplicity of the classifier’s learning algorithm is a crucial aspect. In additional, every time the FSR strap is worn, the force resistive sensors might be positioned in a different location respect to the muscle groups. The classifier has therefore to be retrained every time the strap is worn – high learning speed of the classifier is therefore a desired feature. The ELM was selected to work with the FSR strap for real-time upper-extremities posture classification for its simplicity and fast learning speed.

To evaluate the performance of the proposed system, we developed a test protocol that resembles the sequence of needed steps required to drink from a cup. The drinking task has widely been used in multiple kinds of rehabilitation interventions, including the constraint-induced movement therapy [26]. We discretized the drinking task in a sequence of movement steps, in order to identify at which point the volunteer failed the task. By correctly classifying each step, the FRS strap can potentially provide feedback to the patient to enhance her/his motivation or to the therapist to assess the patient’s improvements. For example, in constraint-induced movement therapy, the patient is required to repeat an exercise a number of times. The quality of the movement gradually worsens with the number of repetitions because of fatigue. By assessing each movement step, the system can identify at which point the volunteer fails to correctly perform the task. Thus, by classifying the intermediate steps, the system is able to provide feedback to help the patient to maintain the quality of the exercise, as well as to provide more detailed information to the therapist for analysing the progress of the rehabilitation.

The proposed work is innovative from different perspectives. Differently from the work proposed in the literature, we used a portable and minimalistic FSR array to capture FMG patterns of the forearm, which enables us to distinguish complex upper limb posture that involves multiple joint movements. The proposed system is also very simple to be worn and used; for instance, the muscle location is not needed to be identified before placing the FSR strap. This work presents real-time FMG classification, which, to the best of the authors’ knowledge, has not been analysed or presented in previous works. The use of real-time FMG classification through the FSR strap is proposed in the interesting task of classifying arm postures during the well-known drinking task.

## Methods

### FSR strap and its placement

**Figure 1:**
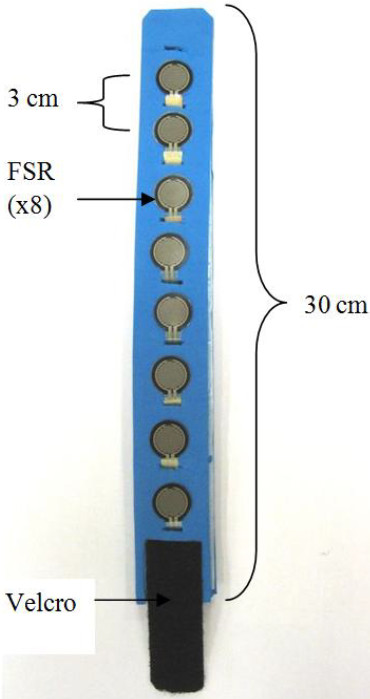
Interior view of FSR strap.

A force sensor resistor (FSR) is made of a polymer thick film that decreases in resistance when pressure is applied onto its sensing area. Eight 0.5′ circular FSRs made by Interlink Electronics [27] were inserted onto a strap made with FloTex foam; the FSR sensors were placed 3 cm apart from each other. The total length of the FSR strap was 30 cm. Velcro tapes were attached on both the interior and exterior end of the FSR strap to secure the strap onto user’s forearm. The interior view of the FSR strap is shown in the Figure 1.

**Figure 2:**
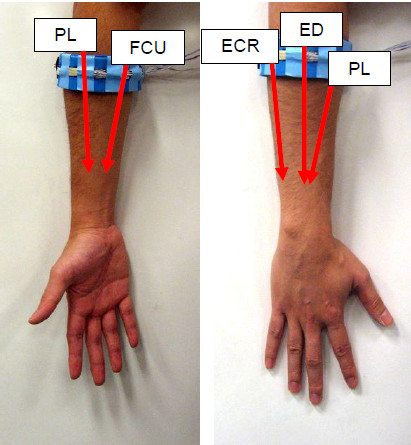
**Placement of the FSR strap.** Red arrow indicates the proximate span of the corresponding muscle.

The FSR strap was designed to be a simple device which can be worn without or with little assistance. The user does not require having muscle physiological knowledge in order to identify the location for the strap placement. He/she can simply wrap the FSR strap around the proximal portion of the forearm, and tight it up with Velcro. The amount of pressure needed to be applied to record FMG is mild, and with the flexibility of the FloTex foam, the FSR strap does not block blood circulation or constrain motion. There are two main reasons for placing the FSR strap on the proximal portion of the forearm (see Figure 2). Firstly, this portion of the forearm covers multiple major muscles groups that control both the hand and wrist movements [13,14], which include, but are not limited to, the Extensor Carpi Radialis, the Extensor Digitorum, the Flexor Carpi Ulnaris and the Palmaris Longus. Secondly, it is also able to detect pattern associate with the elbow movement.

### Data acquisition setup

**Figure 3:**
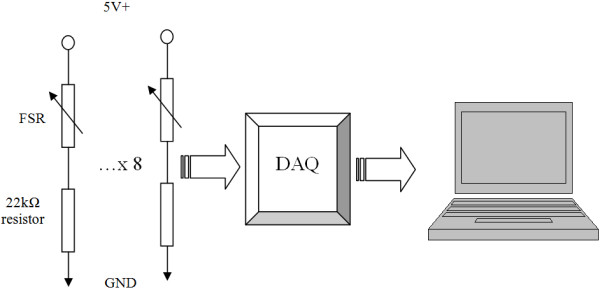
System diagram.

The hardware that was used for extracting the FMG signals was very simple. This aspect represents an advantage of FMG over EMG signals for detecting movements of the upper extremities. Specifically, voltage dividers were used for extracting the signals from the force sensors as shown in Figure 3. In absence of pressure, the resistance of the FSR was more than 10 M Ohm, and it decreased logarithmically as pressure increased. The suitable output range for muscle pressure sensing depends on the base resistor, which was empirically set to be at 22 k Ohm for optimal measurements. The voltage divider circuit was powered by a 5 V voltage source of a data acquisition (DAQ) device made by National Instrument (NI USB 6210). The outputs of the voltage dividers were fed into the DAQ, which was connected to a battery powered notebook computer for signal processing.

### Pattern recognition with non-kernel based extreme learning machine (ELM)

In order to distinguish different upper-extremities postures based on the FSR strap data, we utilized the ELM for real-time classification. Two types of ELM were proposed in [24], the non-kernel based ELM and the kernel based ELM. In this work, we implemented the non-kernel based ELM as its performance is less subjected to the user specified parameters [25]. The non-kernel based ELM has an output function as the following:

(1) $$f\left(x\right)=h\left(x\right)\beta$$

where ***h*** (***x***) is the hidden-layer output corresponding to the input samples from the 8 FSR (***x*** *ϵ* ***R***^8^), and ***β*** is the output weight vector between the hidden layer and the output layer. For multiclass classification, the predicted class label is the index number of the output node that has the highest value.

In equation (1), the hidden-layer output function ***h*** (***x***) maps ***x*** from its original space into an L-dimension space, where L is the number of the hidden nodes, which is specified by the designer. The ***h*** (***x***) has the following form:

(2) $$h\left(x\right)=\left[h\left(a_{1},b_{1},x\right)\ldots h\left(a_{L},b_{L},x\right)\right]$$

where *h*(*a*_*i*_, *b*_*i*_, *x*) is a nonlinear piecewise continuous function with *i* ranging from 1 to L. The parameter *a*_*i*_ and *b*_*i*_ of *h*(*a*_*i*_, *b*_*i*_, *x*) can be randomly generated according to any continuous distribution. Once generated, they can be reused as long as the number of input features and number of hidden nodes do not change. The choice of *h*(*a*_*i*_, *b*_*i*_, *x*) is large, for example it can be a sigmoid, Gaussian, sine, hard-limit, triangular or the radial based functions.

Back to equation (1), the output weight ***β*** is computed based on the following:

(3) $$\beta =H^{T}{\left(\frac{I}{C}+HH^{T}\right)}^{-1}T$$

where ***H*** is the hidden-layer output matrix, ***T*** is the 1-of-K representation of the target label for the training data, and *C*  is the regularization parameter that needs to be specified. ***H*** is constructed from the entire collection of hidden-layer output functions for all hidden nodes and training samples, and it has the following form:

(4) $$H=\left[\begin{array}{c} h\left(x_{1}\right)\\ \vdots \\ h\left(x_{N}\right) \end{array}\right]=\left[\begin{array}{ccc} h_{1}\left(x_{1}\right) & \ldots & h_{L}\left(x_{1}\right)\\ \vdots & \ddots & \vdots \\ h_{1}\left(x_{N}\right) & \ldots & h_{L}\left(x_{N}\right) \end{array}\right]$$

where N is the number of training samples.

In this work, the ELM was implemented in MATLAB for offline analysis and in LabVIEW for real-time classification. There were many choices for  *h*(*a*_*i*_, *b*_*i*_, *x*), however, since the purpose of this paper is not to compare different classifiers’ performance, only the sigmoid function was used. The sigmoid based *h*(*a*_*i*_, *b*_*i*_, *x*) was implemented as the following:

(5) $$h\left(a_{i},b_{i},x\right)=\frac{1}{1+\exp\left(-\left(a_{i}\cdot x+b_{i}\right)\right)}$$

With the hidden-layer output function decided, the number of hidden node (*L*) and the regularization parameter (*C*) were then selected empirically. The common practice for selecting the application dependent parameters, such as L and C, is to use cross validation technique when each time the classifier is trained; however, this approach requires large amount of computation, so it is not suitable for our portable system. Fortunately, the performance of the non-kernel based ELM is not very sensitive to the choice of the parameters *L* and *C*[25]. By increasing the value of L, the classification accuracy increases until it reaches plateau; after that, little improvement can be gained. By choosing a large value for L, high accuracy is guaranteed. However, the larger the L is, the more memory is required. The selection process for the parameter C is different; a large value does not guarantee to have good performance. However, a suitable value for C can be chosen within optimal range. The optimal range of C was empirically found to be between 2^5^ and 2^11^ for this application. Due to the fact that the suitable value of L and C do not have to be unique, there is no need to use cross validation technique, which allows the classifier to be quickly trained. The selected values of L and C were respectively 200 and 2^7^, and they were used throughout the experiment for all the participants.

### Experiment

**Figure 4:**
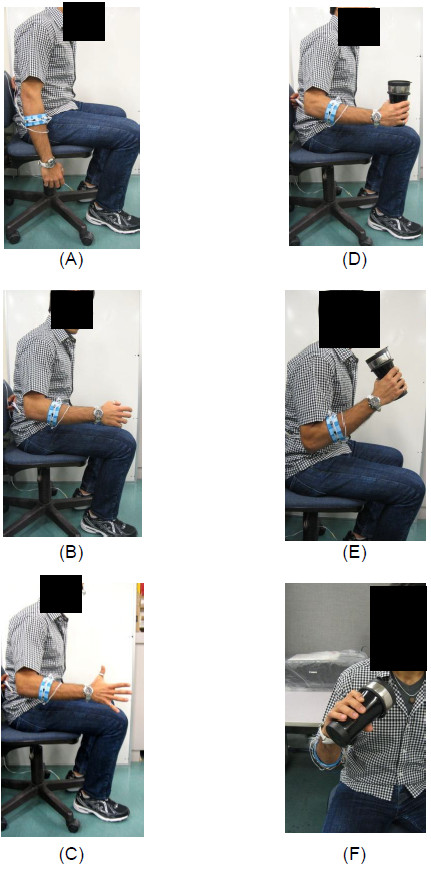
**Classes definition for drinking task postures. (A)** Class 1 - Relax; **(B)** Class 2–90 deg Elbow Flexion; **(C)** Class 3 - Fingers Extension; **(D)** Class 4 – Soft Grasp; **(E)** Class 5 – 120 deg Elbow Flexion; **(F)** Class 6 – Wrist Pronation.

An experiment was designed to evaluate the performance of the combined use of the FSR strap and ELM classifier for upper-extremities posture classification in real-time. A total of six classes were included, and each class corresponding to one distinct posture for the drinking task as shown in Figure 4 (the volunteer gave consent for publication of this image). The postures were associated with the movement, such as rising the forearm (elbow flexion), grasping or releasing the cup (fingers flexion/extension), and repositioning the cup to mouth (wrist pronation). Due to the fact that the FSR strap was only able to monitor the force pressure distribution pattern of the muscles in the forearm, no posture that involved change of shoulder position was included in the experiment. The entire experiment was divided into two phases, training phase and testing phase.

During the training phase, the participant was asked to recreate the six postures shown in Figure 4, and maintain each posture for 7 seconds. During this period, the operator instructed the custom made LabVIEW application to record 5 seconds of data. The entire sequence was repeated 3 times during this phase.

**Figure 5:**
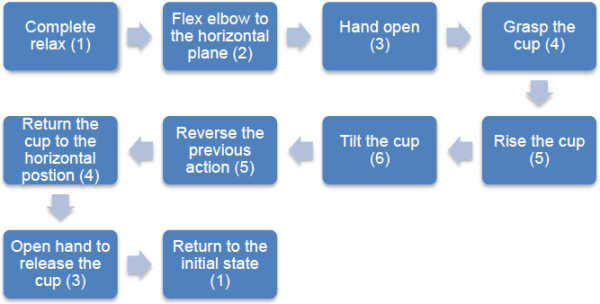
Testing protocol for real-time classification.

During the testing phase, the participant was asked to follow a set of predefined instructions on a monitor to perform the corresponding postures. The instruction sequence with the corresponding class labels (in bracket) is shown in Figure 5 for better understanding the protocol. To start the test, the participant was asked to sit on a chair with upper-extremities completely relaxed (see Figure 4A). Next, he/she raised the elbow to the horizontal plane and then fully extended all the fingers in order to grasp a cup in the next step (see Figure 4B). An empty cup was grasped (Figure 4C) and then the elbow was further flexed (see Figure 4D). In order to resemble the drinking action, the participant tilted the cup toward the mouth with wrist pronation (see Figure 4E). At the end of this task, the volunteer was asked to reversed the actions, namely to supinate the wrist, extend the elbow, extend the fingers, and fully relax the arm. There were therefore a total of 10 instructions (see Figure 5), and each instruction lasted 3 seconds, except for the Class 1 instruction. The participant was asked to repeat the sequence 3 times during this testing phase.

**Figure 6:**
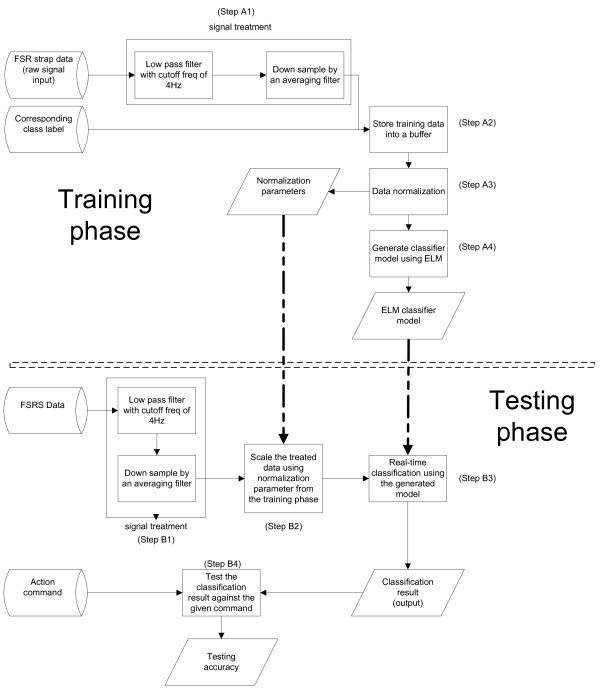
Data processing sequence.

The data processing sequence of the experiment is shown in Figure 6. In both training and testing phases, the FSR strap data was sampled at 1 kHz and passed through a low pass filter with a cut-off frequency of 4Hz to remove high frequency noise. It was then down sampled by an average filter with a moving window of 200 samples and a step size of 100 samples (see Step A1 and Step B1 in Figure 6). During the training phase, 50 samples (corresponding to 5 seconds of data) were collected for each posture, with 6 different postures and 3 repetitions, a total of 900 samples were collected. Each FSR strap sample and the corresponding class label were stored in a training buffer (see Step A2 in Figure 6). Once the training protocol was completed, the entire dataset was saved and then went through a normalization process (see Step A3 in Figure 6) before being used for ELM classifier model generation (see Step A4 in Figure 6). The learning was completed in less than 3 seconds, which allowed the participant started testing without waiting.

During the testing phase, the FSR strap data went through the same filtering process and then was scaled down using the normalization parameters obtained during the training phase (see Step B2 in Figure 6). The scaled sample was classified in real-time (see Step B3 in Figure 6). The classification result along with the processed FSR strap sample and the action command (visual command provided to the volunteers) were recorded for analysis (see Step B4 in Figure 6). Note that even though the prediction was performed in real-time, there was no feedback for the participant; only the operator could see the instant (less than 200 ms delay) classification results.

A total of six healthy male volunteers, who signed an informed consent form (project approved by the Office of Research Ethics, Simon Fraser University), participated in the study. Their average age was 29.7 years old, and the average circumference of their forearm, at the location in which the FSR strap was placed, was 27 cm. The characteristic of the participants are detailed in Table 1.

**Table 1:** Participant statistics

	***Age***	***Proximal forearm circumference (cm)***
Participant 1	24	26
Participant 2	31	27
Participant 3	27	27
Participant 4	35	27
Participant 5	34	27
Participant 6	27	28
Average	29.7	27.2
STD	4.4	0.8

## Results

### Training dataset analysis

**Figure 7:**
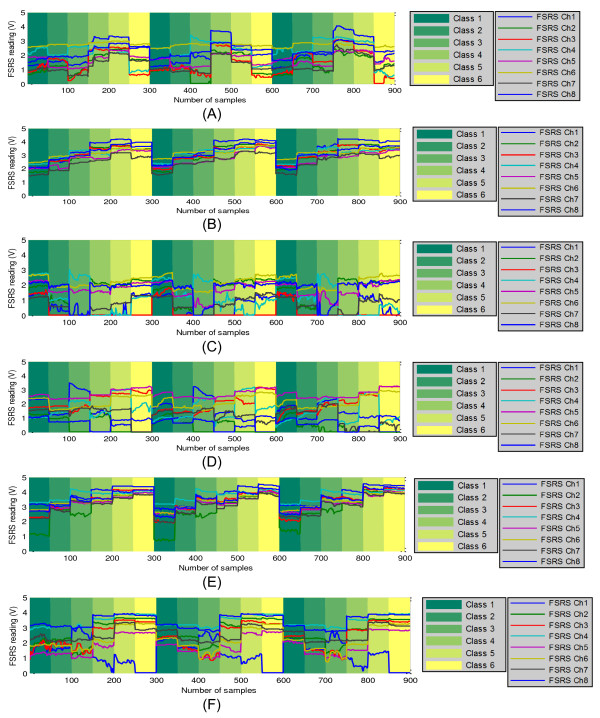
**Training dataset with 50 samples (5 seconds) per class per section. (A)** Dataset for Participant 1; **(B)** Dataset for Participant 2; **(C)** Dataset for Participant 3; **(D)** Dataset for Participant 4; **(E)** Dataset for Participant 5; **(F)** Dataset for Participant 6.

The training data for the 6 participants are plotted in Figure 7. For each dataset, the corresponding class labels are shown with different background colours, and the corresponding training FSR strap data are shown with coloured solid lines. By visually inspecting Figure 7, we can see that the FMG patterns of each participant are different - no obvious trend can be observed. This behaviour was a consequence of the different positions the force resistive sensors had on the muscle groups of each volunteer. Both the different shape and size of the volunteers’ forearms and the different muscle synergies also contributed to have very different signal patterns from one individual to another. This result corroborates the work done by Liarokapis et al. [28] on sEMG analysis, which indicates the model generated by the classifier should be subject-specific.

Figure 7 also shows that the FMG pattern of the same participant varied slightly among the three repetitions that were performed. Besides variations due to the different amounts of force applied by the participant during the different repetitions, FMG pattern changes were also caused by the small displacements of the FSR strap on the forearm during the movements of the upper extremity. Figure 7 shows that the training of the classifier has taken these small variations into account.

### Real-time classification result analysis

**Figure 8:**
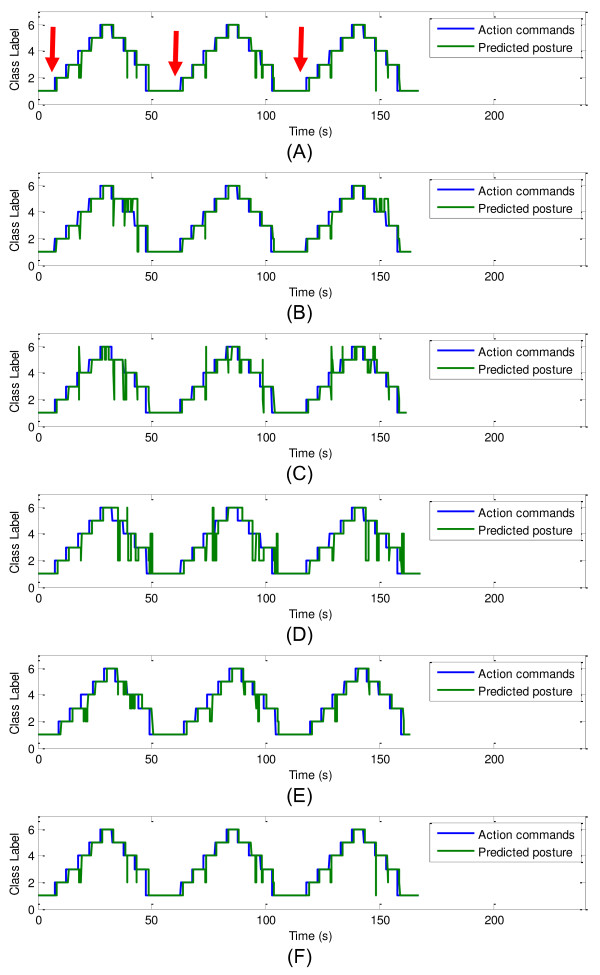
**Real-time classification result. (A)** – Result for Participant 1 with red arrows indicate the transition from Class 1 to Class 2 posture; **(B)** – Result for Participant 2; **(C)** – Result for Participant 3; **(D)** – Result for Participant 4; **(E)** – Result for Participant 5; **(F)** – Result for Participant 6.

The FSR strap data along with the action commands (visual command provided to the volunteers) and the real-time predictions were recorded during the testing phase for off-line analysis. The action commands and real-time predictions for all participants are shown in Figure 8. For each subplot in the Figure 8, the action command sequence is shown with a blue solid line, and the real-time prediction is shown with a green solid line. The action command sequence and the prediction were matched in general, despite a small delay between the two. The delay of the real-time prediction was mainly due to the participant’s response time to the given commands. That is, when a new command was given, the user needed 0.5 seconds to 1.5 seconds to respond, which mostly depended on the participant’s concentration during the test. A measure of the response time for each participant was computed and reported in Table 2. Its calculation was based on the average delay during the transition from Class 1 to Class 2 posture, which is indicated by the red arrows in Figure 8A. Because the volunteers’ response time relatively consistent, this specific transition was selected for the evaluation for the delay. In addition, there was almost no misclassification during this transition for all participants. The average delay for all participants for this transition in each section was computed to be 1 second with an average standard deviation of 0.18 second.

**Table 2:** Time delay for action response

	***Section 1 (s)***	***Section 2 (s)***	***Section 3 (s)***	***Average (s)***	***STD (s)***
Participant 1	1.9	1.0	1.1	1.33	0.49
Participant 2	0.8	1.0	0.7	0.83	0.15
Participant 3	0.8	0.7	0.8	0.77	0.06
Participant 4	1.3	1.5	1.1	1.30	0.20
Participant 5	1.1	1.0	0.9	1.00	0.10
Participant 6	0.7	0.8	0.8	0.77	0.06
Average				1.00	0.18

In order to have an accurate quantitative measure of the proposed method, the delay in time response of each volunteer to the visual commands should be compensated. By shifting the real-time classified output data of each participant forward in time according to the average delay found in Table 2, the real-time testing accuracy was obtained. The accuracy was calculated based on the number of correctly classified data point over the total number of data point. The result is presented in Table 3. This table also reports the most misclassified output data (second column of Table 3) and their corresponding accuracy (third column in Table 3). The overall average classification accuracy for all participants was 92.33% with a standard deviation of 3.19%. The most misclassified output data corresponded to Class 2, namely to the “90 degrees elbow flexion” (see Figure 4B). The average accuracy for the most misclassified output data was above 75%. Based on the statistical data, we concluded the ELM was able to accurately extract the pattern in real-time from the FSR strap for the drinking task.

**Table 3:** Real-time test result

	***Real-time classification accuracy in%***	***Class label of most misclassified output data***	***Accuracy of the most misclassified output data in%***
Participant 1	95.50	3 & 5	93.50
Participant 2	92.20	5	78.20
Participant 3	90.20	2	78.50
Participant 4	87.50	2	50.70
Participant 5	92.70	2	76.40
Participant 6	95.90	2	79.70
Average	92.33		76.17
STD	3.19		13.94

An off-line analysis was also performed to study the general performance of the ELM for different randomly generated hidden-layer bases. Five random hidden-layer bases were generated and applied on to the recorded FSR strap data. The data processing procedure was the same for both the real-time and off-line classification. The result of off-line classification is shown in Table 4. Including the real-time test result, the average accuracy was calculated along with the corresponding standard deviation for each participant. Five out of six participants’ results had standard deviations less than 1.3%, and the result of the remaining one (Participant 4) was 2.4%. The average test accuracy for Participant 4, namely 84.8%, was the lowest amongst all the volunteers. In addition, the real-time accuracy for most misclassified output data (Class 2 data) for the same participant was 50.7% (see Table 3). These data suggested that Participant 4 had complex features in the FSR strap pattern that the ELM was not able to pick up during the test. In order to gain an insight on the reasons of the misclassification, an in-depth analysis about the data acquired from the Participant 4 was performed.

**Table 4:** Offline test result with randomly generated base for ELM

	***Real-time recorded classification result (%)***	***Classification result with random base 1 (%)***	***Classification result with random base 2 (%)***	***Classification result with random base 3 (%)***	***Classification result with random base 4 (%)***	***Classification result with random base 5 (%)***	***Average (%)***	***STD (%)***
Participant 1	95.5	95.5	95.6	95.1	95.5	95.5	95.5	0.2
Participant 2	92.2	93.8	90.0	91.2	91.9	91.3	91.7	1.3
Participant 3	90.2	91.7	88.1	91.2	91.1	90.2	90.4	1.3
Participant 4	87.5	87.3	82.8	85.7	82.2	83.1	84.8	2.4
Participant 5	92.7	92.2	91.0	92.0	91.1	90.9	91.6	0.8
Participant 6	95.9	93.1	93.6	94.1	95.5	93.1	94.2	1.2

### Analysis for data misclassification

**Figure 9:**
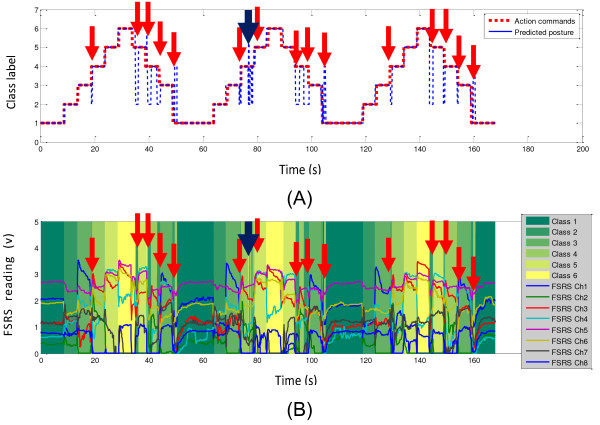
**Real-time test profile for Participant 4.** Red arrows indicate the transitional misclassifications and deep blue arrow indicates the non-transitional misclassification. **(A)** - Real-time Test Result; **(B)** – Real-time Test Data.

The real-time test profile for Participant 4 is shown in Figure 9. In Figure 9A, the red dotted line indicates the class label for the action commands and the blue line indicates the class label for the shifted predicted actions. Figure 9B shows the FSR strap data for all 8 channels with the predicted class labels in the coloured background for easy identification. The red arrows in Figure 9 indicate where the misclassified outputs for Class 2 data occurred. As shown by the red arrows, the majority of the misclassifications occurred during the transitions between two classes and these transitional patterns were mislabelled as Class 2 data. This type of transitional misclassification finds its justification on the training procedure that was used in this study. Specifically, the classifier was trained by using only data recorded outside the transitions. Therefore, it is reasonable to expect misclassification may occur during transitions. Results reported in Figure 9 also show that sometimes misclassification also occurred while the participant was maintaining a steady posture. As indicated by the deep blue arrow in Figure 9, a misclassification was for example found during the Class 4 command, namely “soft grasp” (see Figure 4D), in the second repetition. By analysing the raw data of this specific case in Figure 9B, it can be observed that the FSR strap reading changed while the action command remained unchanged. This behaviour could be attributed to possible variations in muscle recruitment strategies and the nonlinear time response of the FSR sensors.

### Limitations and future work

The scope of the current work is limited to discrete classification of different postures of the volunteers’ upper-extremities. While this approach can be used to provide valuable information to patients and therapists, future work will address classification of continuous movements using FMG signals. The main challenge for implementing this feature is the need of automatically identifying the signature of different types of multi-joints movements involved in a functional task.

## Conclusions

Research was performed towards the development of an affordable and easy to use wearable system that processes force myographic (FMG) signals to provide instantaneous feedback (less than 200 ms) about activities of the upper extremities. A novel force sensing resistor strap for the forearm was developed to capture FMG patterns associated to upper-extremities movements. We utilized extreme learning machine (ELM) to extract the FMG patterns. Specifically, the ELM classifier was implemented in LabVIEW to classify in real-time six different upper-extremities postures associated to the six different steps required to drink from cup. Six healthy volunteers followed a test protocol that was designed to resemble the complete sequence of the drinking task. The average real-time testing accuracy was 92.33% with a standard deviation of 3.19%. This result shows that the proposed device and the use of FMG are potentially suitable to provide accurate feedback to the users about functional movements of their upper extremities.

## Competing interests

The authors declare that they have no competing interests.

## Authors’ contributions

ZGX designed the FSR strap prototype, implemented data collection and real-time classification software, performed experiments, analysed experimental result and participated in manuscript preparation. CM supervised the project, contributed to discussions and analysis and participated in manuscript revisions. Both authors read and approved the final manuscript.
